# Social semantics: the organization and grounding of abstract concepts

**DOI:** 10.1098/rstb.2021.0363

**Published:** 2023-02-13

**Authors:** Penny M. Pexman, Veronica Diveica, Richard J. Binney

**Affiliations:** ^1^ Department of Psychology and Hotchkiss Brain Institute, University of Calgary, Calgary, Canada, T2N 1N4; ^2^ School of Human and Behavioural Sciences, Bangor University, Bangor LL57 2AS, UK

**Keywords:** semantic memory, social brain, embodied cognition, abstract concepts, socialness

## Abstract

Abstract concepts, like *justice* and *friendship*, are central features of our daily lives. Traditionally, abstract concepts are distinguished from other concepts in that they cannot be directly experienced through the senses. As such, they pose a challenge for strongly embodied models of semantic representation that assume a central role for sensorimotor information. There is growing recognition, however, that it is possible for meaning to be ‘grounded’ via cognitive systems, including those involved in processing language and emotion. In this article, we focus on the specific proposal that *social* significance is a key feature in the representation of some concepts. We begin by reviewing recent evidence in favour of this proposal from the fields of psycholinguistics and neuroimaging. We then discuss the limited extent to which there is consensus about the definition of ‘socialness’ and propose essential next steps for research in this domain. Taking one such step, we describe preliminary data from an unprecedented large-scale rating study that can help determine how socialness is distinct from other facets of word meaning. We provide a backdrop of contemporary theories regarding semantic representation and social cognition and highlight important predictions for both brain and behaviour.

This article is part of the theme issue ‘Concepts in interaction: social engagement and inner experiences’.

## 1. Introduction

You are mistaken, Mr Darcy, if you suppose that the mode of your declaration affected me in any other way than as it spared me the concern which I might have felt in refusing you, had you behaved in a more gentleman-like manner.

– Jane Austen, *Pride and Prejudice* [1]

This brief extract from *Pride and Prejudice*, a classic tale in the importance of personal character, integrity and morality, is rich with references to concepts of a social nature (e.g. *manner*, *gentleman* and *refuse*). Indeed, a large portion of even the most everyday vocabulary is occupied by abstract words imbued with a sense of socialness. Arguably, this reflects the vital role of social conceptual knowledge in navigating our interpersonal world. After all, humans are intrinsically and uniquely social. We exhibit a natural propensity to cooperate, coordinate and learn from one another, and to a very large extent, this is done though the medium of language. It is argued that our advanced social cognitive and emotional abilities, as well as the evolution of language, are an adaptation to, and thus a direct consequence of life lived in groups [2,3]. By extension, this suggests there could be a fundamental nature to the social qualities of words.

Recent work in the field of cognitive science has begun to elucidate the ways in which socialness impacts the structure of concepts and the representation of semantic knowledge in the human brain, and this work will be the subject of the first two parts of this paper. In Part A, we will begin with a brief overview of general theories of semantic memory, with a particular emphasis on what is known as the grounding problem and the difficulties it poses for representing abstract word knowledge. Then we will introduce nascent theories that posit social experience as a mechanism for grounding conceptual knowledge, together with a review of recent semantic feature generation/ratings studies that identify socialness as an important factor for distinguishing among different ‘types’ of concepts. In Part B, we will review a set of neuroimaging studies that have approached socialness from a different methodological perspective, exploring if and how socialness of concepts is represented at the level of macroscale brain anatomy. This includes evidence that is in line with claims that social concepts have a special, or even privileged status over other types of concepts, and suggests socialness drives the functional organization of neurobiological systems.

Moreover, a key aim of this paper is to highlight major outstanding questions, and this includes one very fundamental issue that arises from the work described in both Parts A and B: what is it exactly that defines a word as being ‘social’? In Part C, we will discuss the limited extent to which there is consensus on the kinds of semantic features that amount to ‘socialness’ and the degree to which it has been established as a valid and meaningful construct. Consequently, we argue that to further progress theory, the field must first establish a clearer working definition of socialness. To this end, we describe preliminary data from a large-scale rating study in which Diveica *et al.* [4] provided participants with an inclusive definition of socialness and asked them to collectively rate over 8000 English words. This includes findings that appear to confirm that these ratings capture aspects of word meaning that are distinct from those measured via other semantic variables like concreteness.

The issue of whether social concepts are a distinct type, either from other forms of abstract concept or even more generally speaking (i.e. such that this extends to concrete social concepts), has important theoretical implications regarding the fundamental organizational principles underpinning semantic representation.^1^ In turn, it has implications for our understanding of the configuration of brain systems, including those responsible for language and social cognition. These implications extend to applied areas of research where an improved framework for understanding the way social and affective concepts are learned, represented and impaired, could have important implications for educational and clinical practice (see [5,6]).

## 2. Part A – Abstract word representation: a role for socialness?

There are now numerous theories of semantic knowledge, which vary in the extent to which sensorimotor information (e.g. visual, auditory or tactile experience) plays a role in the representation and processing of word meaning. At one end of the spectrum, amodal theories posit that semantic knowledge is represented symbolically, distinct from the ways we experience the world (e.g. [7,8]). At the other end, strongly embodied theories posit that knowledge is represented by sensory and motor systems (e.g. [9,10]). Between these poles lie multimodal or multiple representation theories, which posit that semantic knowledge is represented in many ways (e.g. via language, emotion, introspection and sensorimotor systems), and some versions of those theories include an intermediary supramodal hub (e.g. [11,12]). The hub accounts position language information as one of many types of knowledge connected to the hub. Further, the multiple representation accounts assume that different kinds of information are important for different types of concepts (e.g. [13,14]).

Proponents of semantic theories that include reliance on sensorimotor systems have argued that these theories have the advantage of addressing the grounding problem [15,16]. The grounding problem asks, in essence, if knowledge is represented as symbols, then how do those symbols map to the world? Embodied theories solve that problem by proposing that cognition engages modal systems (e.g. those used for perception, action) to represent semantic knowledge. Strongly embodied theories, however, run into difficulty explaining representation of abstract words. The meanings of abstract words, by definition, cannot be learned or experienced through sensorimotor systems, so they cannot be accounted for by embodied theories. To explain knowledge of abstract words, other means of learning and representation must be considered. Barsalou *et al.* (e.g. [17,18]) have noted that too many approaches to abstract concepts emphasize what they do not contain (sensorimotor information) and that a more positive approach is needed to explore what they do contain. To that end, Barsalou & Wiemer-Hastings [18] (see also [19]) used a property generation task to compare the features of a small set of abstract and concrete words. They found that abstract words were notably different in that their meanings were mainly associated with introspections and, in particular, social aspects of situations, such as people, communication and social institutions.

In addition, work has begun to identify concept ‘types’ within the abstract realm. Much of this work is inspired by multiple representation views and considers multiple sources of grounding beyond the sensorimotor, including the potential contributions of action, language, interoception, emotion, cognition and other internal states. Notably, Borghi and colleagues have proposed the Words as Social Tools (WAT) account, which focuses particularly on the acquisition and representation of abstract word meaning [14,20]. They argue that abstract words are associated with richer linguistic, inner and, importantly for present purposes, *social* experience, than are concrete words (also [21]). Further, they suggest that there could be different types of abstract concepts which vary in their reliance on these different types of information. They suggested that these types of abstract concepts might include institutional, temporal, mental states, emotional, numerical and social concepts.

In related empirical work, researchers have explored the features or properties of abstract word meanings in order to derive potential clusters or types. For instance, Harpaintner *et al.* [22] examined the features listed for 296 abstract words and found that they fell into three clusters. The largest cluster was primarily distinguished by a higher proportion of sensorimotor features, with some social features. A second cluster was distinguished by a high proportion of internal/emotional features and more social features than either of the other clusters. The third, smallest cluster was distinguished by a high proportion of verbal association features. Similarly, Troche *et al.* [23] investigated the organization of abstract and concrete English nouns by asking participants to rate 200 concrete and 200 abstract words on 12 dimensions. They analysed the ratings and identified three latent semantic factors: affective association/social cognition, perceptual salience and magnitude (also see [24]). Abstract word meanings relied more heavily on affective association/social cognition than did concrete meanings. Villani *et al*. [25] asked participants to rate 425 abstract Italian nouns on 15 dimensions and identified four clusters: philosophical/spiritual concepts; physical, spatio-temporal and quantitative concepts; emotional/inner state concepts; and self and sociality concepts. Additional analyses showed that the involvement of the dimension they called social metacognition (defined as a reliance on other people to understand the meaning) distinguished abstract from concrete words, with more abstract words tending to have higher ratings of social metacognition. In addition, ratings on a dimension that they termed social valence (defined as evocative of social situations) were associated with emotion ratings, and with ratings of mouth movement and hearing. These latter relationships were attributed to the important role that language is assumed to play in representing abstract concepts, and to the importance of mouth movement and hearing to language.

Similar conclusions about the existence of types of abstract concepts were drawn from an fMRI study reported by Vargas & Just [26]. They investigated the clustering of 28 abstract words in terms of neural signatures after participants were scanned while thinking of properties of each word. Results showed that there tended to be commonalities across participants in terms of the neural signatures of each word, and the authors identified three latent factors including verbal representation, externality/internality and social content (also see [27,28]).

Thus, there is evidence from some property-generation and feature-rating studies that social words may be a distinct type of abstract word, consistent with assumptions of the WAT theory and other multimodal accounts. Each of these studies, however, has involved a relatively small sample of abstract words, many fewer than people actually know. Therefore, it is possible that the results could be specific to the words tested and may not generalize to a larger set. Thus, there is a need to explore socialness at a much larger scale and right along the concreteness continuum. There is also a need to investigate behavioural effects of socialness. That is, if social words are a distinct type, then one might expect that a word's degree of socialness would be reflected in some way in behavioural measures of lexical-semantic processing, as much as semantic dimensions like valence [29] and concreteness [30] are related to such processing (e.g. [31–33]). One might also expect behavioural responses to social abstract words to be different to those given to other types of abstract words (see [34] for an example of this approach). And yet, comparisons between social abstract words and other abstract word types have still to be made in the context of larger scale behavioural studies. However, they have been contrasted in the neuroimaging literature, reviewed next.

## 3. Part B – Socialness and the brain

A review of neuroimaging literature concerning the representation of abstract concepts identified a small number of papers that treat social concepts *a priori* as a discrete ‘category’ [35]. Most of these studies contrasted social words with a more general class of abstract or concrete words and set out to identify common activity, and/or that which is uniquely associated [36–39]. The earliest of these studies generated a hypothesis that social concepts are a class of concepts with a special, or even privileged status over other types of conceptual knowledge [36,37]. In this context, social conceptual knowledge has been broadly defined as person-specific knowledge [40], but also knowledge about interpersonal relationships, social behaviours and of more abstract social concepts such as *truth* and *liberty* [36,41]. These early studies revealed a patch of anterior temporal association cortex that the authors claimed is selectively involved in processing semantic information of a social nature [40,41].

The ‘social knowledge hypothesis’ [36,41] can be likened to other forms of ‘multiple semantics’ views [42–44] in which the semantic system is composed of multiple independent stores that are differentiated by their link to distinct sensorimotor modalities. Of course, the difference is that the social distinction is based on domain-specificity rather than modality. To understand how this hypothesis formed the starting point for this particular set of neuroimaging studies, one can look to the broader social neuroscience field from which they stemmed. The emergence of this field was triggered, at least in part, by the ‘social brain hypothesis’ [45,46], which states that the expansion of frontal and temporal neocortices across primate species in the human evolutionary lineage is explained by their increasingly high levels of sociality (see [47] for a related review). This created a pervasive assumption, sometimes implicit, that there is a circumscribed set of brain regions that are dedicated to, and, by inference, support specialized processes for social perception and cognition [45,48]. The extent to which domain-specificity of systems for processing social information exists is hotly debated [49–52], but there is evidence for the existence of brain regions or pathways that are sensitive to socialness, particularly at the level of perceptual processes [53–55]. This includes visual cortex with ostensibly selective engagement by faces [56], bodies [57] and social interactions [58]. Whether this putative specialization cascades downstream to higher-order cognitive systems (e.g. memory; executive function) is a more contentious issue [52,59–61].

To date, the leading candidate in terms of a locus for a selective social semantic system lies within the dorsolateral aspects of the anterior temporal lobe (ATL), specifically the anterior superior temporal gyri/sulci [41,62,63]. These ATL subregions exhibit elevated blood-oxygen-level-dependent responses when semantic judgements made on socially relevant stimuli are compared to those made on non-social stimuli [36–39,64,65]. The dorsolateral ATL also appears to increase its response in line with an accumulation of social meaning across connected text [66]. The role of the ATL in representing social knowledge has been ascribed with a right lateralization within some accounts [67], although individual fMRI studies [37,38,62,65,68] and meta-analyses [62,63] indicate bilateral involvement (also see [69,70]).

More recent neuroimaging studies have attempted to disentangle the socialness effect driving some ATL activations from other potentially confounding variables. For example, it is possible that the social concepts explored in neuroimaging studies are, on average, more abstract than more general classes of concepts. However, studies have shown that preferential left hemisphere dorsolateral/polar ATL activation cannot be easily explained by differences in concreteness, or at least imageability, between social words and control words [38,64,65,71], nor by differing degrees of multiplicity of single word meanings (sometimes referred to as ‘semantic diversity’ [72]) [38]. A putative involvement of these regions in combinatorial conceptual processes does also not appear to explain differential engagement by social and non-social words [73,74]. Many of these studies have also been careful to rule out explanations in terms of fundamental lexical properties such as word frequency or syllable/word length [38,64,65,68]. Another semantic factor that could covary with socialness, and account for some preferential activations, is emotional valence. Indeed, one study has shown that social-emotional stimuli elicit stronger responses in the left dorsolateral/polar ATL than other social words, which in turn activate the region more strongly than stimuli lacking any social meaning [75]. However, Wang *et al.* [71] demonstrated at least partially dissociable responses across left lateral ATL subregions to the socialness, valence and abstractness dimensions underlying word meanings. Overall, this collection of neuroimaging studies suggests that the socialness of a concept makes a unique contribution to driving differential recruitment of the brain regions involved in processing meaning (for neuropsychological evidence, see [76,77]). Moreover, they are to some extent compatible with the claim that there is a semantic system dedicated to the representation of social conceptual knowledge, and that this is located in the left dorsolateral/polar ATL [36,40,41].

Of course, an alternative to the notion of a ‘social brain’ or, more precisely, that there are networks or subsystems specialized for social processes, is that social cognition is underpinned by a set of domain-general systems [49,51,52]. As alluded to above, from a strong version of this perspective, socialness effects at the levels of brain and behaviour reflect variations among more general properties of stimuli and/or task demands, rather than socialness *per se*. From a more compromising perspective, it is argued that social interaction could draw on an array of neurocognitive systems in something of a unique way, but, fundamentally, those systems are built for more generalized processes (e.g. [51,52]). For example, an alternative to domain-specific accounts of ATL function like the social knowledge hypothesis is the ‘graded semantic hub’ account proposed by Binney *et al.* [38,78–80]. According to this framework, the whole ATL comprises a unified semantic representational space, all of which is engaged by the encoding and retrieval of concepts, and by concepts of any kind. At the centre of this space lies the ventrolateral ATL, which has a supramodal semantic function, meaning that its engagement during semantic processing is invariant to, for example, idiosyncratic task features, including the modality through which concepts are accessed. Near the edges of this space, however, there are connectivity-driven gradual shifts in semantic function toward subspecializations for processing certain types of semantic features (for a computational exploration of this general hypothesis, see [81]). This might include, for example, at the dorsolateral aspects, a specialization for processing socio-emotional semantic features [38], which could arise from greater proximity and connectivity to the limbic system [78,81,82] (also see below). Consistent with this account are a series of neuroimaging studies by Binney *et al.* which show that, when care is taken to ensure that fMRI signal can be acquired from across the whole ATL, it becomes clear that the ventrolateral ATL activates strongly and equivalently during semantic judgements made on social and non-social stimuli [38,39] (also see [80]). This same ventrolateral ATL region is implicated in general semantic processing in several neuropsychological, neurostimulation, neuroimaging and electrophysiological studies that have used a variety of verbal and nonverbal tasks/stimuli [83–90]. Critical for this graded hub account is the additional fact that the omni-category response of the ventrolateral ATL is much greater in magnitude than that of the social-selective response of the dorsolateral ATL [38,39]. Therefore, these latter studies suggest that, at least within the ATL, differences in the way the brain is engaged by social and non-social concepts are small, or subtle, compared to the similarities. This is consistent with the claim that, rather than there being distinct systems for social and general semantic representation, there is a single domain-general conceptual system and parts of this system are dynamically and differentially engaged by different types of meaningful stimuli and semantic task demands (cf. the Social Semantics framework outlined by Binney & Ramsey [52]; also [11]).

The graded hub hypothesis is an extension of the hub-and-spoke model of semantic representation proposed by Patterson *et al.* [11,12]. According to this framework, the ATL sits at the heart of a spoked semantics architecture comprised of association regions involved in modality-specific sensorimotor processing, as well as affective and linguistic processes. The hub-and-spoke model emphasizes that semantic representation arises from the *conjoint* action of modal systems and an intermediary supramodal hub [11]. It offers a reconciliation between distributed-only embodied accounts in which concepts are dependent upon systems involved in sensory and motor processing [91–94] and neuropsychological and computational modelling data that point towards the existence of a hub (e.g. [95,96]). A fuller discussion surrounding the necessity of a hub is beyond the scope of this review, and for a starting point we refer the reader to Lambon Ralph *et al*. [11], as well as Meteyard *et al*. [44]. However, we have chosen to raise this broader hub-and-spoke proposal here because it is a neurobiologically constrained model that, like multimodal or multiple representation views, acknowledges sources of semantic information beyond sensorimotor experience, including contributions from language, emotion and other internal states [11]. Moreover, like some of the multimodal views described in the previous section (e.g. [13,14]), it hypothesizes that different types of concepts (e.g. tools) can vary in their reliance on different sources of information (e.g. object affordances and kinematics), which will be reflected in differential engagement of spoke regions [97,98] (also see [99]). This notion lends one interpretation to neuroimaging studies that investigate social concept representation and implicate brain regions outside of the ATL. For example, two recent studies have demonstrated an apparent selective engagement of the precuneus, a region associated with visual-spatial imagery [100], during the processing of abstract social words [26,101]. This could reflect a tendency for social concepts to draw differentially upon systems that capture visual or spatial elements of interpersonal contexts [26].

## 4. Part C – What is ‘socialness’?

In the sections above we have provided a brief overview of two parallel literatures among which socialness has begun to emerge as an important organizational principle underpinning semantic representation. In Part A, we described property generation and feature rating studies that have explored the attributes of abstract words and have extracted socialness as a latent factor that distinguishes abstract from concrete words, and even distinguishes different types of abstract words. In Part B, we reviewed a literature that has emerged in parallel, describing a set of neuroimaging studies that have probed socialness as a predictor of differential patterns of brain activation evoked during semantic processing. In contrast to property-generation research, most of these neuroimaging studies approached social concepts as an *a priori* discrete type of concept. This has, thus far, been fruitful in that this brain-based evidence points to socialness being independent of more general semantic properties, such as abstractness, emotional valence and other facets of single-word meaning. There is now a burgeoning debate regarding the relative size of the contribution that socialness makes to semantic representation. On one hand, it has been argued that social words are a distinct type and, moreover, that there are specialized neural systems underpinning social semantics. On the other hand, socialness can be framed as one of many dimensions that coexist to define a single representational space underlying general semantics.

However, we assert that, while these lines of research are both intriguing and promising, the conclusions and discussions that have transpired from them are mostly premature because the ostensive evidence has accumulated in the absence of clear boundaries between what is social and what is not. This is true both at the level of theory and in the empirical measures. Without agreeing on this definition, at least to some extent, it will not be possible to compare theories and evaluate evidence in support of them. So, what is socialness actually?

Socialness as a construct has been characterized variably in terms of behavioural descriptiveness, and social concepts have been distinguished from non-social concepts on divergent sets of criteria. To illustrate this point, we have collected examples in table 1 (also see [35]). Many of these studies focused on a word's reference to social interaction by measuring, for example, the extent to which a word refers to relationships between people [23,24], or how often its referent involves interaction between people [64,65,71,74]. By contrast, other definitions emphasize specific aspects of social experience, such as how well a word describes social behaviours [36], or the degree to which word meanings relate to the relationship between self and others [104].

**Table 1.** Definitions used to measure socially relevant semantic constructs in previous studies.

publication	name of construct	type	definition
Arioli *et al*. [102]	sociality	dimension	how much a word inherently refers to information concerning social as opposed to individual contexts
Binder *et al*. [103]	social	dimension	the degree to which one thinks of a thing as an activity or event that involves an interaction between people
	human	dimension	the degree to which one thinks of a thing as having human or human-like intentions, plans or goals
	communication	dimension	the degree to which one thinks of a thing as a thing or action that people use to communicate
	self	dimension	the degree to which one thinks of a thing as related to your own view of yourself, a part of your self-image
Catricalà *et al*. [70]	social	dimension	how much a word is linked to a social situation or to an interaction among people, both in terms of inclusion and exclusion
Crutch *et al*. [104]	social interaction	dimension	the degree to which concepts relate to the relationships between self and others
Diveica, *et al.* [4]	socialness	dimension	the degree to which a word's meaning has social relevance by describing or referring to a social characteristic of a person or group of people, a social behaviour or interaction, a social role, a social space, a social institution or system, a social value or ideology, or any other socially relevant concept
Harpaintner *et al.* [22]	social constellation	category	a feature or a situation that describes the coexistence of different persons or which implies an interaction between at least two different persons
Lin *et al*. [68]	sociality	dimension	the number of people involved in an event to which a verb refers
	biological motion	dimension	the extent to which the meaning of a verb brings to mind biological motion
Lin *et al*. [64,65,74], Wang *et al.* [71]	sociality	dimension	how often the meaning of a word/the use of an object involves an interaction between people
Mellem *et al*. [75]	social content	category	referring to people either by a proper name or the name of an occupation/title
Roversi *et al.* [105]	institutional objects	category	an artefact that performs its function via the collective acceptance displayed by a given community (status function) and not in virtue of its physical features
	social objects	category	an entity that presupposes the existence of at least two agents engaged in some form of common activity and that does not have a clear status function attached to it
Troche *et al.* [23,24]	social interaction	dimension	the degree to which the word relates to relationships between people
	morality	dimension	the degree to which the word relates to morality, rules, or anything that governs one's behaviour
Vargas & Just [26]	social content	dimension	the degree to which the concept involves social interaction or self-perception as affected by social interaction
Villani *et al*. [25]	social metacognition	dimension	how much others were needed to understand the meaning of the word
	social valence	dimension	the degree to which the concept evokes social circumstances
Villani *et al*. [106]	pure institutional objects	category	entities constituted by formalized rules in a social framework
	meta-institutional objects	category	concepts that are necessary to define the content of institutions but are not defined by those institutions
Zahn *et al*. [36]	behaviour descriptiveness	dimension	how well a word describes a detailed specific set of social behaviours of persons
	social category breadth	dimension	how many different kinds of social behaviours of persons a word can apply to
Zhang *et al*. [66]	social semantic richness	dimension	the extent to which the word/sentence/narrative is related to interactions between people

Following a review of the material presented in table 1, we suggest that there are two distinct emerging approaches to the construct of socialness. On one hand, there are social measures designed to capture contextual information, such as the degree to which a word meaning evokes a set of social circumstances [25], or whether it applies to social as opposed to individual contexts [102]. On the other hand, there are measures probing specific social features of word meaning, such as the scale of interaction/number of agents implicated [68] and the degree to which a referent has human-like intentions, plans or goals [103]. This distinction might reflect different representational frameworks for meaning, such as those based on features/similarity and those based on association [107,108], and it could be an important avenue for future research into the mechanisms by which socialness is attributed to concepts. However, the heterogeneity in definitions across this set of studies is striking, highlights theoretical inconsistencies, and hinders our ability to compare findings across studies. Certainly, it imposes grave limitations on the conclusions that can be made presently about socialness as a neurobiologically and/or behaviourally relevant principle.

We argue that, to further progress theory, the field must first establish a clearer working definition of socialness. Further, the field would be advanced if large-scale norms of rated socialness were available, much as they have been made available for thousands of words for semantic variables like concreteness [30], emotional valence [29] and others. We believe this can best be achieved, at least initially, by adopting a broad definition of socialness. To aid this endeavour, we recently obtained ratings for 8388 English words by asking participants to rate socialness according to the following definition [4]:

the extent to which each word has social relevance by describing or referring to a social characteristic of a person or group of people (e.g. ‘trustworthy’), a social behaviour or interaction (e.g. ‘to fight’), a social role (e.g. ‘teacher’), a social space (e.g. ‘pub’), a social institution (e.g. ‘hospital’) or system (e.g. ‘nation’), a social value (e.g. ‘righteousness’) or ideology (e.g. ‘feminism’), or any other socially-relevant concept.

**Figure 1.**
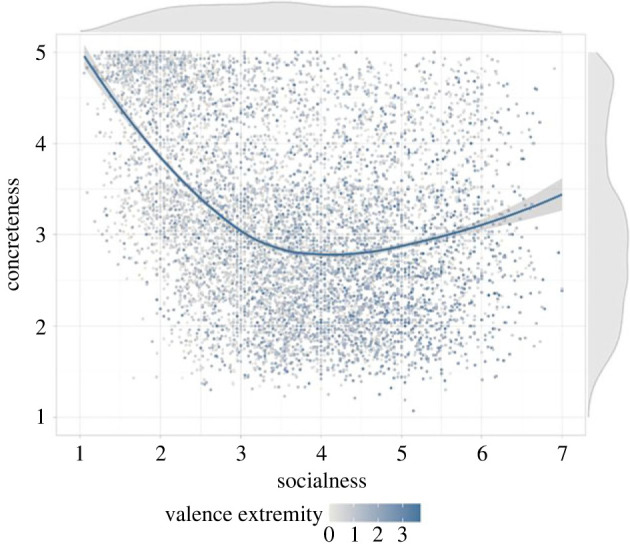
The relationship between socialness ratings [4] and concreteness ratings [30] for 8388 English words is illustrated and highlighted by the loess line. The colour of the dots represents valence [29] extremity—the darker the colour, the more valenced the word. The density distributions of the socialness and concreteness dimensions are plotted on the top and right of the graph, respectively. The graph shows that words with high mean socialness ratings span the entire concreteness dimension, and that the socialness measure captures information distinct from valence.

To our knowledge, the resulting norms are the largest set of openly available word socialness ratings. We believe that employing an inclusive definition was a crucial next step for understanding the construct of *socialness*. This allowed us to test the extent to which socialness is reliably perceived as a broad construct, and as applicable to various types of words/parts of speech. Initial explorations of the ratings reveal that, when broadly defined, socialness ratings have good reliability and validity. We have begun exploring to what extent these new socialness ratings capture aspects of word meaning that are distinct from those measured via other semantic variables, such as concreteness and emotional valence (figure 1). Results showed that socialness is negatively correlated with concreteness [30], but also that the two variables share only a modest 10% of variance. Another key observation was that words rated as high in socialness spanned the entire concreteness dimension, from concrete concepts like *people* and *festival* to abstract ones like *democracy* and *cooperate*. As might be expected [23], socialness was positively associated with valence extremity (the absolute difference between the valence rating and the neutral point of the original valence scale [29]), but it shared only 4.8% of variance. We provide more extensive description and exploration of the socialness norms in Diveica *et al.* [4] but, in summary, our preliminary analyses indicated that this socialness measure captures a distinct psycholinguistic construct.

## 5. Conclusion and future directions

The research we have reviewed here suggests that socialness, broadly construed, is a dimension of word meaning that can be distinguished from other dimensions such as concreteness and valence. Moreover, there is some evidence that socialness is reflected within the organization of neural systems that support semantic processing. It remains to be seen whether this is indicative of social words being a distinct type, or whether socialness is just one of many dimensions that define a unified domain-general semantic space. At present, there remain two key shortcomings in this exciting area of research. First, researchers need to begin agreeing on terms and definitions of ‘socialness’ so that we are better able to compare theories and evaluate evidence in support of them [109]. Second, there is very little research on the behavioural consequences of socialness and behavioural relevance is, of course, a gold standard for psychological theory. In terms of refining models of semantic representation, we believe that there are four key avenues for future research, and they have been made possible by the availability of the new socialness ratings [4] described above. We will outline these research questions in the paragraphs below.

First, there are testable predictions that can be derived from WAT and other multiple representation theories. For instance, WAT proposes that social experience is key to learning and representing abstract concepts [14]. In line with this proposal, one could predict that (i) socialness facilitates the acquisition of abstract words, (ii) socialness contributes to the acquisition of abstract words more than to that of concrete words and (iii) abstract words are associated with more social content than are concrete words. In addition, WAT proposes a close link between linguistic and social experience. Consistent with this, Villani *et al*. [25] found that, in a sample of abstract words, the more the words evoked social circumstances, the more they relied on auditory experiences, and on mouth motor system activation. These relationships could be further evaluated to understand how linguistic and social information jointly support acquisition and representation of abstract words.

Second, Diveica *et al.* [4] characterized socialness in a broad and inclusive way and found this to be a useful and meaningful starting point. However, subsequent research is needed to more thoroughly explore the nature of the information captured by the socialness dimension and to evaluate whether there are important distinctions that it does not capture. In future research, it will be helpful to consider narrower definitions to explore whether there are clusters or subtypes of social words. Moreover, it remains to be seen what aspects of the social experience, such as those measured by the more specific socially relevant dimensions listed in table 1, are most related to lexical-semantic processing in terms of both behaviour and brain. It is possible that there are sub-types of social words that rely on different kinds of information. To some degree, this could mirror the more general concrete-abstract distinction, possibly in terms of how concepts rely differentially upon qualitatively different representational frameworks, such as those based on features/similarity and those based on association (cf. the proposal outlined by Crutch and Warrington [107,108]). For example, Roversi *et al.* [105] investigated the properties associated with two potential sub-types of social concepts. They found that ‘social objects’ (defined in table 1), such as *choir*, elicited mainly contextual situations (e.g. *concert*), while institutional artefacts, such as *marriage*, evoked a higher proportion of normative relations (e.g. *commitment*). Further, the abstract-concrete distinction was more marked for social objects compared to institutional artefacts. Social objects that are concrete were associated with thematic/situational relations, while those that are abstract elicited more mental associations. In a related study, Villani *et al*. [106] proposed a further distinction between pure institutional concepts (e.g. *marriage*) that relied more on exteroceptive information, and meta-institutional concepts (e.g. *duty*) for which interoceptive, affective and metacognitive information was more important. Future research that applies a data-driven approach across a large sample of abstract and concrete words will shed light on more specific socialness constructs and the way in which individual social word meanings potentially cluster together into sub-types.

Third, there are several implications for neuroimaging research into the representation of social concepts, and we have some recommendations. Now that large-scale socialness ratings are available and their independence from measures of concreteness and emotional valence has been more firmly established [4], researchers are better positioned to comprehensively disentangle the neural correlates of socialness from other semantic variables. Indeed, right across the line of neuroimaging research reviewed in Part B, there is a need for greater integration of the kind of property generation, feature rating and behavioural research we reviewed in Part A. It will be instrumental for driving the next set of key questions, including those regarding the neural correlates of different types of concepts, and a putative privileged status afforded by socialness [52]. At present, there is a lack of clear evidence in favour of an absolute boundary between social concepts and other types of concepts, and this suggests that there is going to be considerable overlap in the systems that represent them [38,39]. In this case, it will be important to use experimental designs and analytical techniques that allow for detecting more graded distinctions. To date, socialness has only been explored using univariate, magnitude-based approaches, whereas information-based approaches, including multivariate pattern analysis and repetition suppression paradigms, will be essential, particularly for understanding overlapping activation, which could reflect either shared processes or tightly yet separately packed cognitive functions that only dissociate when investigated at higher spatial resolutions [110,111]. Moreover, a key methodological determinant for obtaining a complete picture of the neural basis of social concepts will be the use of neuroimaging techniques that maximize the signal obtained from across the entirety of key brain regions. This includes the anterior temporal lobe, of which some subregions are invisible to standard fMRI [38,80,112].

Fourth, it is worth noting that concepts are not static and that their representation depends on ongoing task contexts as well as prior experience [113,114]. For example, it has been shown that concepts are to some degree influenced by culture and the language spoken [115,116]. Given that cultural environments are intrinsically linked to our social experiences, social concepts might be particularly susceptible to cultural influences. Moreover, a variety of socially relevant characteristics (e.g. race, gender) impact our social experiences, which can consequently lead to between-individual variability in the representation of social concepts. In line with this, Mazzuca *et al*. [117] showed that the features participants associated most strongly with the social concept *gender* depended on their gender identity and sexual orientation. This potential variability could be investigated in future research and might manifest in various ways. For instance, some abstract words, including those high in social content, might place greater demands upon cognitive control processes because their exact meaning is dependent on context. This might be reflected in differential engagement of regions associated with controlled semantic selection and retrieval, such as the left inferior frontal gyrus (IFG; see [118–120] for related discussions). Consistent with this, some individual neuroimaging studies reported greater activation of semantic control regions (the IFG) during the processing of social, as compared to non-social words/sentences [38,75] (also see [121]). In addition, there is some limited behavioural evidence that implicit semantic processing of social words compared to non-social words slows reaction times in a Stroop task in adults [102] and in a selective attention task in children [122], indicating greater demand for cognitive control. However, more research is needed to understand what task contexts and concept features might drive an increased need for regulatory mechanisms when processing social concepts.

In summary, in the present paper, we have outlined the ways in which two different literatures have explored the idea that social concepts might be a special type and have offered suggestions for integrating and advancing these research efforts. Further, we presented initial psycholinguistic explorations of a new and openly available set of socialness ratings for over 8000 words (see [4] for a detailed description). These suggest that socialness is indeed a distinct aspect of word meaning and one that should be incorporated in theories of semantic representation. Social words, like *manner*, *gentleman* and *refuse*, convey information about our relationships with people and inform our understanding of their actions. Socialness gives words salience and gives meaning to the interactions and events that make up sources like *Pride and Prejudice*, and that occur in the personal and interpersonal complexities of our everyday lives.

## Data Availability

The data illustrated in figure 1 are available via the Open Science Framework at https://osf.io/2dqnj/.

## Footnotes

1 There is some debate about whether the conceptual system is separate from the lexical-semantic system. We take the position that the conceptual system is not separate from the meaning accessed during language processing.
